# Closed Reduction and Percutaneous Pinning in Isolated Cuboid Dislocation Management: A Case Report

**DOI:** 10.7759/cureus.49023

**Published:** 2023-11-18

**Authors:** Rachel A Loyst, Frederick Hance, Megan Paulus

**Affiliations:** 1 Orthopedic Surgery, Stony Brook University, Stony Brook, USA

**Keywords:** closed reduction, surgical case reports, foot and ankle, percutaneous pinning, cuboid dislocation

## Abstract

Cuboid dislocations are a rare type of injury with few cases reported. A 41-year-old female came in for an assessment of her left foot, seeking evaluation 13 days post-injury. On inspection of the left lower extremity, we found swelling and ecchymosis throughout the midfoot. There was dimpling along the fourth/fifth tarsometatarsal (TMT) joint with palpable dorsal subluxation. A closed cuboid reduction with percutaneous pinning was performed 20 days after the initial injury. The cuboid was reduced with a combination of traction and direct pressure. One 1.6 mm Kirschner wire was passed from the fifth metatarsal across the TMT joint into the cuboid. At the 10-week follow-up appointment, she was ambulating with her boot and had successfully returned to work as a teacher. Radiographs demonstrated a maintained reduction of the dislocation and interval healing of the navicular and fourth metatarsal base fractures. Dislocations of the cuboid have only a handful of cases reported. They can occur in isolation or with other injuries of the midfoot. This patient was successfully treated with closed reduction and percutaneous pinning. Further studies are required to obtain a consensus on optimal treatment for these types of injuries.

## Introduction

Cuboid dislocations are a rare type of injury with few cases reported. The dislocation of the cuboid bone typically requires significant damage to the surrounding ligaments, and many of the documented cases occurred during traumatic events such as motor vehicle accidents [1-4]. Due to the complex articulations in a two-dimensional view, dislocations of the tarsal bones can be easily overlooked in radiographic imaging analysis [4,5]. Inferomedial direction was the most commonly observed direction of cuboid dislocations in the documented case reports [5-7]. Additionally, excessive plantar flexion has been identified as a contributing factor in both cuboid fracture and dislocation [1,8].

## Case presentation

**Figure 1:**
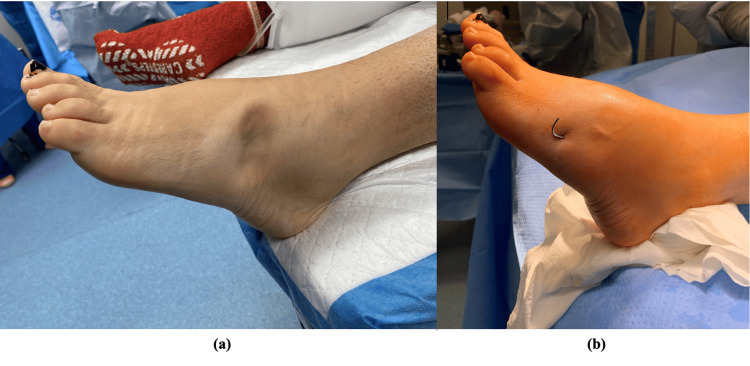
Clinical photo of patient pre-operatively demonstrating puckering of the skin (a) over the lateral midfoot with immediate improvement after pinning (b).

A 41-year-old female came in for an assessment of her left foot, seeking evaluation 13 days post-injury. She described the initial injury as a twisting of the left foot after she stepped off a curb while working as a teacher at school. She had immediate pain and was unable to bear weight. She was seen at an outside hospital and sent to our office for surgical management. On clinical examination of the left lower extremity, we found swelling and ecchymosis throughout the midfoot. There was dimpling along the fourth/fifth tarsometatarsal (TMT) joints along with palpable dorsal subluxation of these joints (Figure 1). Additionally, tenderness along the midfoot from the first through fifth TMT joints, in addition to tenderness over the medial aspect of the navicular, was noted. 

**Figure 2:**
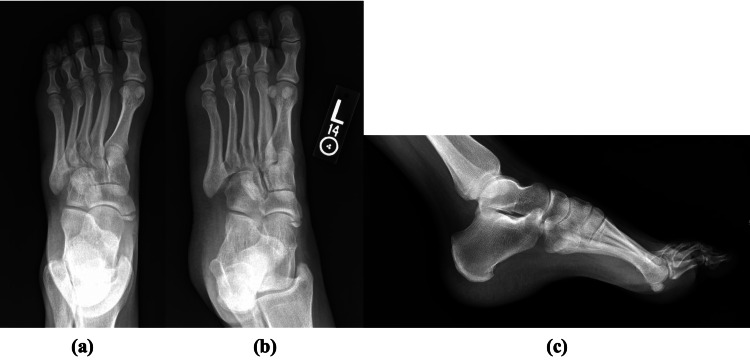
Non-weight bearing anteroposterior (a), oblique (b), and lateral radiographs (c) of the left foot pre-operatively.

**Figure 3:**
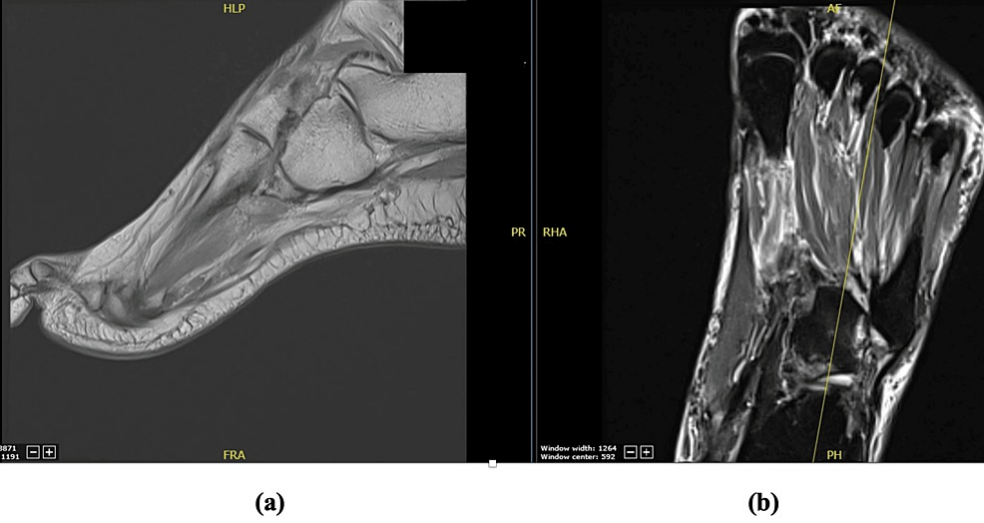
Sagittal (a) and coronal (b) MRI views of the foot pre-operatively demonstrating the inferomedial migration of the cuboid.

**Figure 4:**
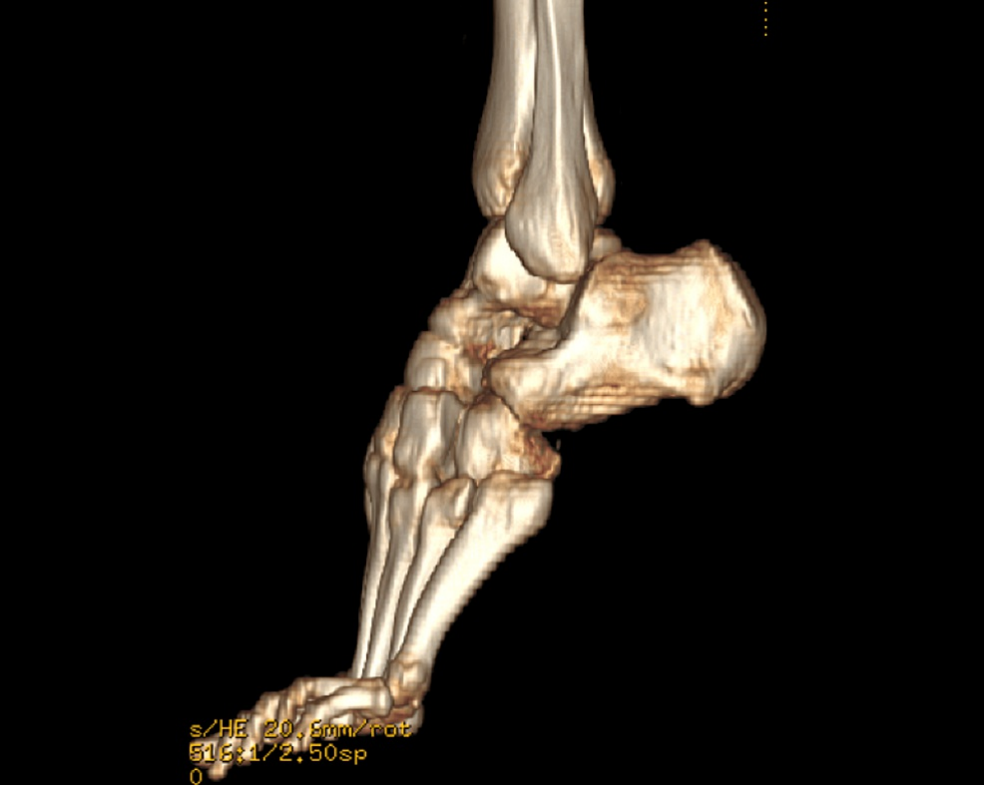
Three-dimensional CT view of the foot pre-operatively demonstrating the dislocation of the cuboid.

Non-weight bearing standard three view X-rays from the outside hospital demonstrated cuboid dislocation inferomedially in relation to the fourth and fifth metatarsals at the TMT joint, with no significant diastases noted at the Lisfranc interval (Figure 2). Further imaging, including MRI and CT of the foot, was also ordered due to the patient being unable to tolerate weight-bearing films. The MRI and CT redemonstrated that the cuboid was dislocated inferomedially in relation to the fourth and fifth metatarsals (Figure 3 and Figure 4, respectively). There were partial tears of the TMT joint capsules. It also revealed nondisplaced intra-articular fractures at the third metatarsal base, the plantar margin of the lateral cuneiform, and a 1 mm depressed fracture on the plantar surface of the first metatarsal base. 

**Figure 5:**
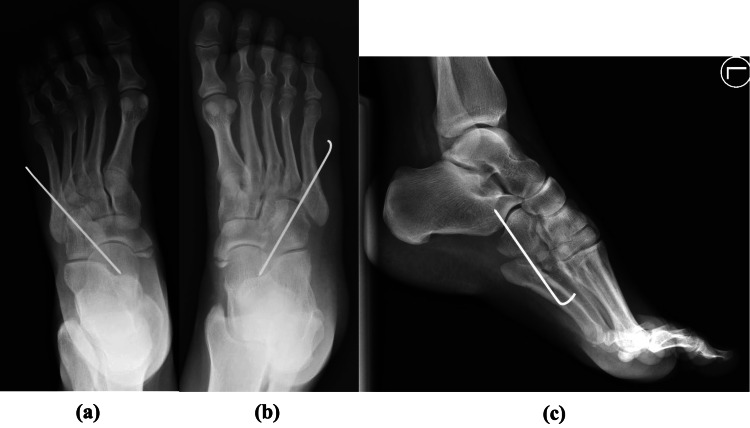
Post-operative anteroposterior (a), oblique (b), and lateral (c) radiographs demonstrating pinning of the fifth TMT joint with reduction of the cuboid. TMT, tarsometatarsal

The patient underwent operative management. A closed cuboid reduction with percutaneous pinning was performed 20 days after the initial injury. The cuboid was reduced with a combination of traction and direct pressure to elevate the cuboid. Once it was reduced, it felt stable and was pinned with the reduction held manually. One 1.6 mm Kirschner wire was passed from the fifth metatarsal across the TMT joint into the cuboid (Figure 5). There was excellent stability following pinning. The navicular and fourth metatarsal base fractures were nondisplaced and were treated non-operatively. The skin puckering seen pre-operatively improved after reduction.

**Figure 6:**
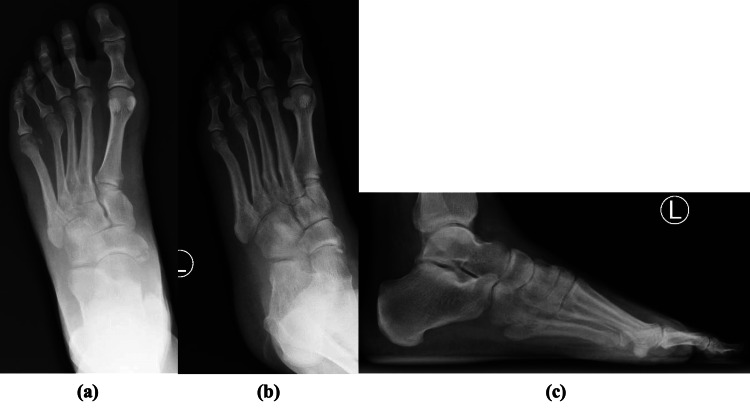
Weight-bearing anteroposterior (a), oblique (b), and lateral (c) radiographs at the 10-week follow-up appointment.

Post-operatively the patient was made non-weight bearing in a short leg AO splint. The pin was removed at six weeks. At this point, the patient was made weight-bearing as tolerated in a pneumatic walker. At the 10-week follow-up appointment, she had been walking with her boot and was able to return to work as a teacher. Radiographs demonstrated a maintained reduction of the dislocation and interval healing of the navicular and fourth metatarsal base fractures (Figure 6). She felt that her pain had improved but still had mild pain with activity. She was instructed to continue weight bearing as tolerated and wean herself out of the pneumatic walker. 

## Discussion

The cuboid articulates with five bones, and it is not surprising that dislocations are uncommon [1,6]. Cuboid dislocation can be treated operatively with open reduction and temporary wire or screw placement [3,4]. Most patients make a full recovery with a return to baseline [4]. Cuboid dislocations are not usually managed with closed reduction, as only one successful case was reported [9].

The anatomic relationship of the cuboid with other bony and ligamentous attachments is why the cuboid is very difficult to injure, especially in isolation. The cuboid articulates with both the TMT joints (Lisfranc joint) and midtarsal joints (Chopart joints) [4]. Strong dorsal and plantar ligamentous attachments stabilize the cuboid proximally, medially, and distally to the calcaneus, lateral cuneiform and navicular, and fourth and fifth metatarsal bases [4]. In this case, there was no obvious ligamentous damage on MRI. 

The best surgical treatment for cuboid dislocations has not yet been determined because of the rarity of this injury. Rigid internal fixation with plates and screws and percutaneous pin fixation have both been used [4,6,10]. Opting for rigid fixation could be favored to achieve a more stable construct, but temporary pinning might be preferred to reduce the risk of joint damage and stiffness in the lateral mobile column. There is no consensus in the literature regarding this injury so the fixation method remains surgeon-dependent. 

We opted to temporarily pin the dislocation to preserve the motion of the lateral column in this young, active patient. In this case, open reduction was not necessary. However, this is not typical for this type of injury. Intra-operative fluoroscopy demonstrated excellent alignment, and the dislocation was found to be quite stable. Had this not been the case, we would have opted to open the fracture for reduction and pin it with one or multiple Kirschner wires. 

## Conclusions

Dislocations of the cuboid are rare injuries with only a handful of cases reported. They can occur in isolation or with other injuries of the midfoot. This patient with isolated cuboid dislocation was successfully treated with closed reduction and percutaneous pinning. Further studies are required to obtain a consensus on optimal treatment for these types of injuries. 
